# A deep network embedded with rough fuzzy discretization for OCT fundus image segmentation

**DOI:** 10.1038/s41598-023-27479-6

**Published:** 2023-01-06

**Authors:** Qiong Chen, Lirong Zeng, Cong Lin

**Affiliations:** 1 College of Electronic and Information Engineering, Guangdong Ocean University, Haida Road, Zhanjiang, 524000 Guangdong China; 2 Department of Earth System Science, Tsinghua University, Shuangqing Road, Beijing, 100084 Beijing China; 3 School of Information and Communication Engineering, Hainan University, Renmin Avenue, Haikou, 570228 Hainan China

**Keywords:** Biomedical engineering, Applied mathematics, Computational science, Computer science, Information technology

## Abstract

The noise and redundant information are the main reasons for the performance bottleneck of medical image segmentation algorithms based on the deep learning. To this end, we propose a deep network embedded with rough fuzzy discretization (RFDDN) for OCT fundus image segmentation. Firstly, we establish the information decision table of OCT fundus image segmentation, and regard each category of segmentation region as a fuzzy set. Then, we use the fuzzy c-means clustering to get the membership degrees of pixels to each segmentation region. According to membership functions and the equivalence relation generated by the brightness attribute, we design the individual fitness function based on the rough fuzzy set, and use a genetic algorithm to search for the best breakpoints to discretize the features of OCT fundus images. Finally, we take the feature discretization based on the rough fuzzy set as the pre-module of the deep neural network, and introduce the deep supervised attention mechanism to obtain the important multi-scale information. We compare RFDDN with U-Net, ReLayNet, CE-Net, MultiResUNet, and ISCLNet on the two groups of 3D retinal OCT data. RFDDN is superior to the other five methods on all evaluation indicators. The results obtained by ISCLNet are the second only inferior to those obtained by RFDDN. DSC, sensitivity, and specificity of RFDDN are evenly 3.3%, 2.6%, and 7.1% higher than those of ISCLNet, respectively. HD95 and ASD of RFDDN are evenly 6.6% and 19.7% lower than those of ISCLNet, respectively. The experimental results show that our method can effectively eliminate the noise and redundant information in Oct fundus images, and greatly improve the accuracy of OCT fundus image segmentation while taking into account the interpretability and computational efficiency.

## Introduction

**Figure 1:**
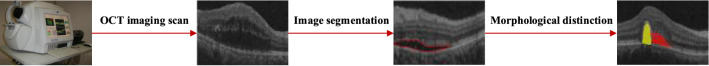
Automatic fluid region segmentation process of OCT fundus images.

The macular region is the most sensitive part of the fundus to light^1^. Once inflammatory reaction and liquid infiltration occur in this area, edema lesions will be formed^2^. Macular edema can cause visual impairment or even visual loss, and is secondary to retinal diseases^3^. Optical coherence tomography (OCT) has the characteristics of high resolution, non-invasive, and non-contact, and has become an indispensable imaging method in the diagnosis and treatment of macular diseases^4,5^. OCT can identify three types of liquid lesions, namely, intraretinal fluid (IRF), subretinal fluid (SRF), and pigment epithelial detachment (PED)^6^. Although OCT has become a standard tool for quantitative analysis of macular diseases in clinic, manual analysis of fluids is subjective, labor-intensive, and error prone^7^. Therefore, automatic fluid region segmentation is proposed to assist ophthalmologists in detecting abnormal diseases of fundus structures^8^. The automatic fluid region segmentation process of OCT fundus images is shown in Fig. 1. First, the OCT image is obtained after being scanned by the OCT device. Then, the automatic segmentation algorithm is used to divide the lesion area in the image, and further distinguish the morphology of the lesion area.

Traditional methods used by early OCT segmentation systems include threshold-based segmentation^9^, graph-based segmentation^10^, segmentation based on the fuzzy level set with a cross-sectional voting^11^, segmentation based on a locally adaptive loosely coupled level set^12^, or machine learning algorithms using manual features^13^. However, these traditional methods are sensitive to image quality and lack generalization ability. Semi-supervised methods can solve the problem of retinal OCT image segmentation with low contrast and speckle noise, but need to rely on expert information for multiple iterations^4^.

As a mathematical method to deal with imprecise, uncertain, and incomplete data, rough set can discover hidden knowledge and reveal potential laws through analyzing and reasoning for data. Since rough set requires no prior knowledge, it has also been applied to medical image segmentation. Banerjee et al. used both rough sets and contraharmonic mean for bias field estimation to remove intensity inhomogeneity artifact from MR images^14^. Jothi et al. used a hybridization of two techniques of tolerance rough set and firefly algorithm to select the imperative features of brain tumor from the segmented MRI images^15^. Although rough set exhibits good performance on medical image segmentation, it is difficult to handle continuous features. In addition, rough set is prone to generate a large number of classification rules, which substantially increases the amount of calculation.

Compared with the above-mentioned segmentation methods, the segmentation methods based on deep learning can automatically learn and extract image features, thus making great improvements^16–22^. Deep learning can not only provide powerful models to represent complex relationships, but also make highly accurate predictions from complex data sources through multi-level structure^23,24^. Ronneberger et al. proposed the U-shape Net (U-Net) framework showing promising results on the neuronal structure segmentation in electron microscopic recordings and cell segmentation in light microscopic images, which has become a popular neural network architecture for biomedical image segmentation tasks^16^. Roy et al. proposed an end-to-end fully convolutional framework (ReLayNet) for reliable segmentation of retinal layers and fluid masses in eye OCT scans^20^. Gu et al. proposed a context encoder network (CE-Net) to capture more high-level features and preserve more spatial information for 2D medical image segmentation^25^. Ibtehaz et al. developed a novel architecture (MultiResUNet) to improve the performance of the U-Net model in segmenting multimodal medical images^26^. He et al. proposed an intra- and inter-Slice contrastive learning network (ISCLNet) to improve the point-supervised OCT fluid segmentation for a rapid and accurate prediction of fluid regions^8^.

Deep learning technology has achieved promising results in OCT fundus image segmentation. However, the presence of noise and redundant information in images is the main reason for the performance bottleneck of deep networks^27–29^. Affected by the quality of imaging sensors, the effect of environmental conditions, and the interference in the transmission channel, OCT fundus images inevitably introduce noise and redundant information in the process of formation, transmission, reception, and processing. Noise and redundant information not only reduce the quality of the original image, but also make errors accumulate among the network layers, which seriously affects the segmentation performance of the deep neural network^30^. As an important data preprocessing technology, feature discretization is widely used in big data analysis^31–33^. It is able to convert continuous attribute values into discrete ones, thereby eliminating the negative effects of noise and redundant information^34,35^. In addition, feature discretization can be useful for missing value imputation, thereby repairing the missing regions in the image^36,37^.

Another problem of deep networks is the lack of robustness and interpretability, and they are difficult to deal with uncertainty information caused by noise, disturbance, and blurred boundary^38–40^. Although feature discretization enables the dimension reduction of complex data and eliminates the negative effects of noise and redundant information, the uncertainty information such as randomness and fuzziness contained in the data makes it difficult to obtain the high-quality discrete intervals. The rough fuzzy set^41^ is considered to be a more powerful model for big data uncertainty analysis than the fuzzy set^42^ and the rough set^43^. By introducing the fuzzy membership into the equivalence relation of the rough set to describe the correlation between samples, it has more flexibility in dealing with uncertain information. The feature discretization based on the rough fuzzy set can obtain the best discretization result by effectively quantifying the uncertain information in the data. Therefore, the combination of deep learning and feature discretization based on the rough fuzzy set provides a feasible solution for improving the segmentation effect of OCT fundus images.

To this end, we propose a deep network embedded with rough fuzzy discretization (RFDDN) for OCT fundus image segmentation. Our main contributions are as follows:

1. we establish the information decision table of OCT fundus image segmentation, and calculate the membership degrees of pixels to each segmentation region using the fuzzy c-means clustering to achieve the fuzzification of pixel categories;
2. we design the individual fitness function based on the rough fuzzy set, and use a genetic algorithm to search for the best breakpoints to discretize the features of OCT fundus images to reduce the uncertainty caused by noise and redundant information;
3. we take the feature discretization based on the rough fuzzy set as the pre-module of the deep neural network, and introduce the deep supervised attention mechanism to obtain the important multi-scale information, thereby improving the segmentation accuracy of OCT fundus images.

We compare RFDDN with the state-of-the-art segmentation algorithms on OCT fundus images. The experimental results show that our method can effectively eliminate the noise and redundant information in Oct fundus images, and greatly improve the accuracy of OCT fundus image segmentation while taking into account the interpretability and computational efficiency.

The rest of this paper is arranged as follows: section “Related work” reviews the related work; section “Methods” elaborates the proposed algorithm flow; the experimental results are analyzed and discussed in section “Results and discussion”; section “Conclusion” summarizes this paper.

## Related work

We introduce the definition of feature discretization and the binary coding method of genetic algorithm, and explain the basic concepts of rough sets and fuzzy sets. Then, we describe the deep supervised attention mechanism.

### Feature discretization and binary coding method

**Figure 2:**
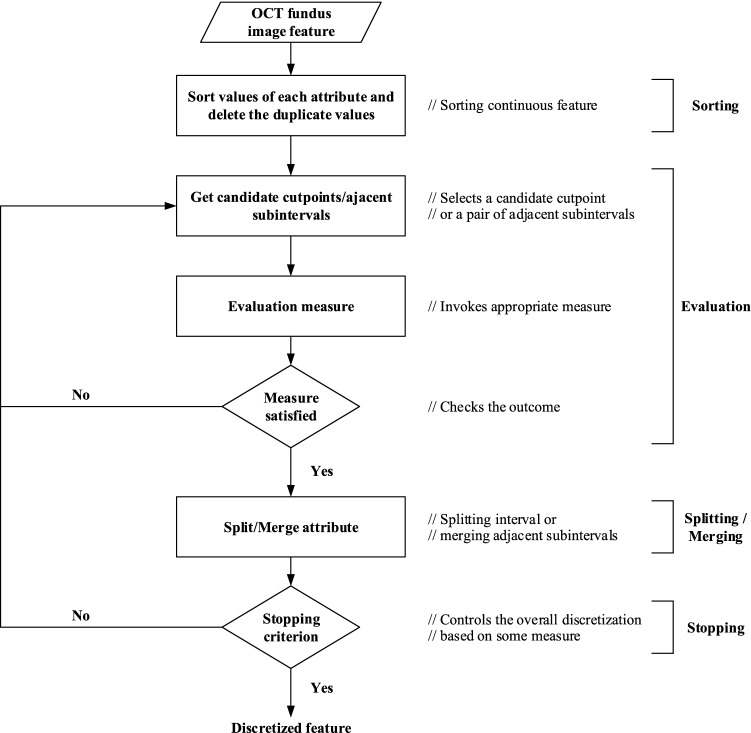
Basic flow of OCT fundus image feature discretization.

In feature discretization, continuous attributes are divided into a finite number of subintervals, and then, these subintervals are associated with a set of discrete values^44–46^. The basic flow of OCT fundus image feature discretization is shown in Fig. 2. First, the pixel values of the OCT fundus image are sorted, and the duplicate values are removed to obtain a set of candidate breakpoints. Second, the breakpoints of continuous attributes are selected from the set of candidate breakpoints, and whether to segment the interval or merge the adjacent subintervals is decided according to the judgment criteria of the adopted discretization algorithm. If the termination condition is satisfied, the discretization result is output; otherwise, the remaining breakpoints are continuously selected from the set of candidate breakpoints to perform attribute discretization.

**Figure 3:**
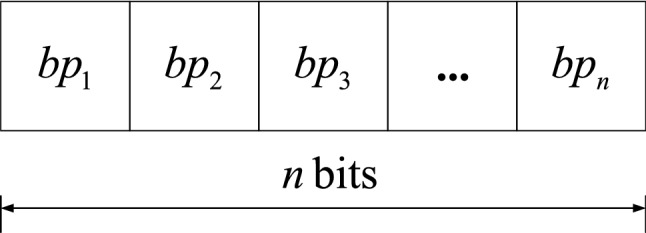
Chromosome structure.

The genetic algorithm has inherent implicit parallelism and strong global search ability, and has achieved promising results on the problem of feature discretization^47^. The genetic algorithm uses binary coding to encode the candidate breakpoints. Each bit in the binary code corresponds to a candidate breakpoint. The values ‘1’ and ‘0’ represent that the breakpoint is selected and discarded, respectively. Assuming that *BP* ($BP=\left\{ bp_1, bp_2, \ldots , bp_n\right\}$) is the candidate breakpoint set of an OCT fundus image, the chromosome structure in the genetic algorithm is shown in Fig. 3. The length of chromosome is *n* bits. The set of selected candidate breakpoints is a discretization scheme.

### Fuzzy set and rough set

A fuzzy set is used to characterize the fuzzy phenomenon that is difficult to measure precisely because of no strict boundary division^42^. A fuzzy set (*A*) is defined as follows:

1 $$\begin{aligned} A=\left\{ \left( x, \mu _A(x)\right) : x \in U\right\} , \end{aligned}$$

where *U* is the universe, and $\mu _A(x)$ ($0 \le \mu _A(x) \le 1$) is the membership of *x* to *A*.

The rough set regards knowledge as the ability to classify objects in the universe^43^. The two-tuple ($K=(U, {\mathbb {R}})$) is a knowledge base, where ${\mathbb {R}}$ is an equivalence relation cluster on the universe (*U*). For *X* ($X \subseteq U$), the lower and upper approximations of *X* with respect to *R* ($R \in {\mathbb {R}}$) are

2 $$\begin{aligned} {\underline{R}} X= & {} \left\{ x \in U \mid [x]_R \subseteq X\right\} , \end{aligned}$$

3 $$\begin{aligned} {\overline{R}} X= & {} \left\{ x \in U \mid [x]_R \cap X \ne \varnothing \right\} , \end{aligned}$$

where $[x]_R$ ($[x]_R=\{y \in U \mid (x, y) \in R\}$) is the equivalence class of *x* under *R*. The quotient set ($U / R=\left\{ [x]_R \mid x \in U\right\}$) is called a knowledge.

The fuzzy set and the rough set represent two different approaches to uncertainty. The fuzzy set addresses gradualness of knowledge by membership function, whereas the rough set addresses granularity of knowledge by equivalence relation. The rough fuzzy set combines the advantages of the rough set and the fuzzy set, and has a more powerful uncertainty analysis capability^48^. The rough fuzzy set can also be combined with deep learning to achieve a more powerful system regarding to learning and generalization. Affonso et al. presented a methodology to biological image classification through a rough-fuzzy artificial neural network^49^. This work shows that combining the deep learning with rough fuzzy sets has a great potential for OCT fundus image segmentation.

### Deep supervised attention mechanism

The attention mechanism exploits the autocorrelation matrix to capture the long-distance dependence between pixels in OCT images, enabling the neural network to focus on the extraction of key features^50^. On this basis, the deep supervised attention mechanism realizes the mapping of the representation of pixels to area objects by constructing the correlation between pixels and area objects.

**Figure 4:**
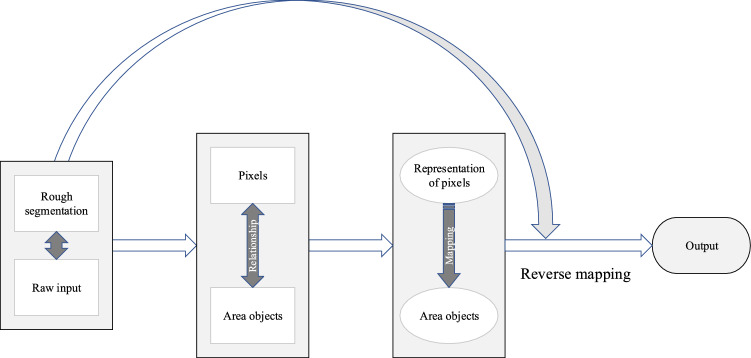
Principle of the deep supervised attention mechanism.

The principle of the deep supervised attention mechanism is shown in Fig. 4. First, the rough segmentation map formed by deep supervision and the raw input are used to construct the relationship between pixels and area objects, thus realizing the mapping of the representation of pixels to area objects. Then, the original spatial features are restored by reverse mapping, thereby enhancing the context representation of pixels to classes.

## Methods

We first introduce the process of calculating membership by fuzzy *c*-means clustering (FCM). Then, we construct the fitness function using the rough fuzzy set as the discretization criterion. Finally, we elaborate RFDDN for OCT fundus image segmentation.

### Membership calculated by FCM

In general, it is prone to errors when an object in a dataset is completely classified into a certain category. FCM can assign a weight between each object and each category to indicate the degree to which the object belongs to the category^42^. To build a rough fuzzy model applied to OCT fundus images, we first need to find out the membership functions of pixels to segmentation regions.

An OCT fundus image can be represented by an information decision table ($S=(U, B, C, V, f)$), where *U* is the pixel set, *B* is the brightness attribute, *C* is the category attribute of the segmentation region, *V* is the range, and *f* is the mapping function from objects to each attribute range. Assuming that *U* contains *N* pixels, the number of categories is *M*, $x_i$ is the brightness value of the *i*-th pixel ($1 \le i \le N$), and the brightness value of the class center of the *j*-th category is initialized to $c_j^0$ ($1 \le j \le M$), then the membership of the *i*-th pixel to the *j*-th category is initialized as

4 $$\begin{aligned} u_{i j}^0=1\Bigg / \sum _{k=1}^M\left( \frac{\left( x_i-c_j^0\right) }{\left( x_i-c_k^0\right) }\right) ^2. \end{aligned}$$

Then, the brightness value of the class center of the *j*-th category is updated in the next iteration as

5 $$\begin{aligned} c_j^1=\sum _{i=1}^N\left( \left( u_{i j}^0\right) ^2 \times x_i\right) \Bigg / \sum _{i=1}^N\left( u_{i j}^0\right) ^2. \end{aligned}$$

The membership and the brightness value of the class center are updated iteratively until the following termination conditions are met:

6 $$\begin{aligned} \max _{i j}\left\{ \left| u_{i j}^{t+1}-u_{i j}^t\right| \right\} <\varepsilon , \end{aligned}$$

where *t* is the number of iterations and $\varepsilon$ is the error threshold. Thus, the membership of each pixel in *U* to each category is obtained, as shown in Algorithm 1.
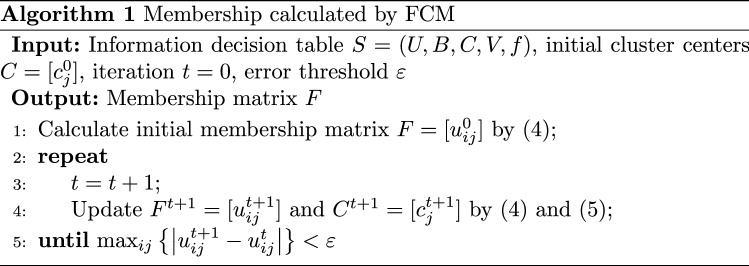


### Fitness function based on the rough fuzzy set

After the membership functions of pixels to segmentation regions are calculated by FCM, we can regard the category of each segmentation region as a fuzzy set and combine the rough set to build a rough fuzzy model of OCT fundus image discretization. The membership function of the *j*-th category is

7 $$\begin{aligned} A_j(i)=u_{i j}, \end{aligned}$$

where $A_j$ is the fuzzy set corresponding to the *j*-th category, and $u_{i j}$ is the membership of the *i*-th pixel to the *j*-th category. The lower and upper approximations of the pixel (*x*) in the rough fuzzy model established by *R*[*B*] and $A_j$ are

8 $$\begin{aligned} R[B]_* A_j(x)= & {} \inf _{y \in U}\left\{ A_j(y) \mid (x, y) \in R[B]\right\} , \end{aligned}$$

9 $$\begin{aligned} R[B]^* A_j(x)= & {} \sup _{y \in U}\left\{ A_j(y) \mid (x, y) \in R[B]\right\} , \end{aligned}$$

where *R*[*B*] is the equivalence relation generated by the brightness attribute (*B*). Correspondingly, the average approximate precision of rough fuzzy sets of all categories is

10 $$\begin{aligned} {\bar{\eta }}=\frac{1}{M} \sum _{j=1}^M \frac{{\text {card}}\left( R[B]_* A_j\right) }{{\text {card}}\left( R[B]^* A_j\right) }, \end{aligned}$$

where $0 \le {\bar{\eta }} \le 1$, and ${\text {card}}(\bullet )$ is the function for calculating the cardinality of fuzzy sets^34^. The larger ${\bar{\eta }}$, the higher the approximation precision. The optimal discretization scheme is the best trade-off between the average approximation precision and the number of breakpoints^41^. Assuming that $N_{DS}$ is the number of breakpoints of the discretization scheme (*DS*) and $N_{CB}$ is the number of candidate breakpoints, the fitness function is designed as follows:

11 $$\begin{aligned} \text{ Fitness } =\alpha \times \left( \frac{N_{C B}-N_{D S}}{N_{C B}}\right) +\beta \times {\bar{\eta }}, \end{aligned}$$

where $\alpha$ and $\beta$ are weight coefficients ($\alpha \ge 0$, $\beta \ge 0$, $\alpha +\beta =1$). We perform genetic operations iteratively to search for the optimal breakpoint set, as shown in Algorithm 2. First, we create membership functions of all categories, and establish the lower and upper approximations of the rough fuzzy set. Then, we design the fitness function based on the average approximate precision and the number of breakpoints. Finally, the individual with the highest fitness is updated to the global variable in each genetic operation. When the precision required by the system is met or the number of iterations set by the user is exceeded, the program is stopped and the optimal discretization scheme is output; otherwise, the genetic algorithm continues to be executed until the termination conditions are met.

The establishment of the rough fuzzy model needs to go through fuzzy *c*-means clustering and the generation of the equivalence relation. The time complexities of these two stages are $O(N \times M \times (N+M) \times t)$ and $O(N^2)$, where *N* is the number of pixels, *M* is the number of categories, and *t* is the number of iterations. Furthermore, it is necessary to calculate the lower and upper approximations of all pixels to obtain the average approximate precision of the rough fuzzy model. As the lower and upper approximations can be calculated simultaneously in one traversal of all pixels, the time complexity of this process is $O(N^2)$. In general, *M* is much smaller than *N* and *t*. Therefore, the time complexity of the rough fuzzy model is about $O(N^2 \times t)$.
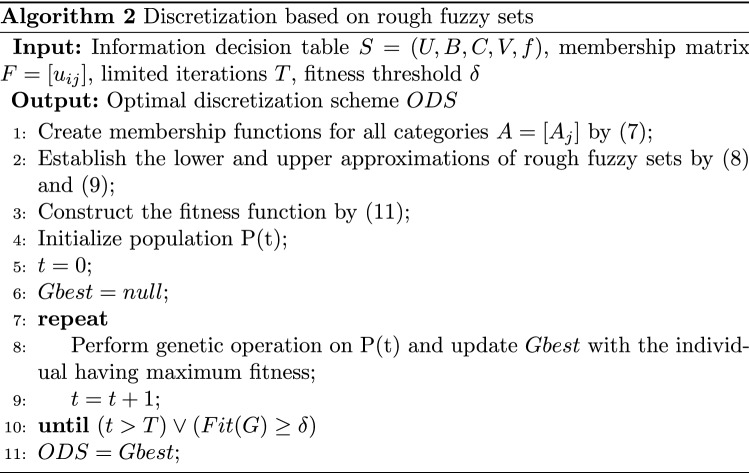


### Overall framework of RFDDN

OCT fundus image is input into the network for processing after the feature discretization based on the rough fuzzy set, and output the final segmentation result. The network of RFDDN adopts an encoder-decoder architecture with forward skip connections from the encoder stage to the corresponding decoder stage. In the process of fusion of shallow features and deep features of the network, we introduce a dual attention mechanism of the spatial region and the feature channel, enabling the deep network to adaptively select the relatively important information from the feature space for fusion. Furthermore, we use the staged attention refinement module to capture multi-scale contextual information through the hybrid kernel convolution. The deep network processes 3D blocks with a size of $64 \times 64 \times 64$. The backbone network uses forward skip links and the residual structure as the basic convolution module of the segmentation model. This structure with skip connections facilitates the propagation of gradient information. The input of each stage is passed through two $3 \times 3 \times 3$ convolution layers, followed by the batch normalization (BN), and prevent overfitting through activation unit (ReLU). We add a residual connection between the input and output of the convolution block. The number of feature maps in the encoder increases with the reduction of feature size, where the minimum number is 16 and the maximum number is limited to 128. Then, the up-sampling size of each feature map is calculated and input into the block. The convolution operation of the kernel is applied to each feature mapping. Accordingly, we create 16 features in each feature mapping. The feature map improves the gradient flow through the deep supervised module. At the same time, the output of the attention module generates feature maps by using the deep supervised module and concatenation. Finally, these feature maps are processed by BN and ReLU after passing through the two convolution layers, thereby generating probability maps of segmentation regions.

**Figure 5:**
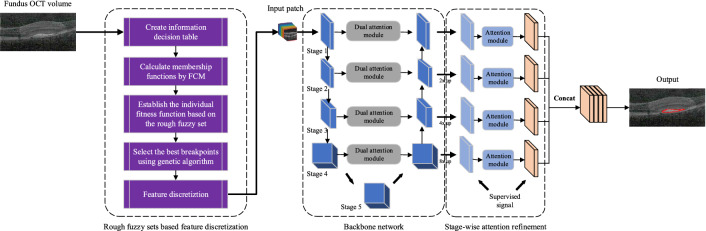
Flow of RFDDN.

The flow of RFDDN is shown in Fig. 5. We take the feature discretization based on the rough fuzzy set as the pre-module of RFDDN. For each input OCT fundus image, the module first creates an information decision table of OCT fundus image segmentation. Then, fuzzy c-means clustering is used to calculate the membership of each pixel to the category of each segmentation region. Finally, the individual fitness function based on the rough fuzzy set is established according to membership functions and the equivalence relation generated by the brightness attribute, and the best breakpoints are selected by a genetic algorithm to discretize the features of the input OCT fundus image. The feature discretization of brightness values of all pixels of the input OCT fundus image can not only remove the redundant information, but also weaken the negative impact of the noise. RFDDN can perform well in complex images affected by noise, disturbance, and lack of clear boundaries. We incorporate the weight information into the cross entropy to optimize the network and alleviate imbalances. The cross entropy loss ($L_{max}$) of the deep supervised branch is defined as follows:

12 $$\begin{aligned} L_{\max }=-\sum _{i=1}^k w_i g_i \log \left( p_i\right) , \end{aligned}$$

where *k* is the number of categories of the segmentation regions, $g_i$ is the gold standard of the *i*-th category, $p_i$ is the prediction probability of the *i*-th category, and $w_i$ is the weight of the *i*-th category. The dice similarity coefficient (DSC) loss ($L_{DSC}$) and the cross entropy loss ($L_{CE}$) are

13 $$\begin{aligned} L_{D S C}= & {} 1-\frac{1}{k} \sum _{i=1}^k \frac{2 g_i p_i+\varepsilon }{\left( g_i+p_i\right) +\varepsilon }, \end{aligned}$$

14 $$\begin{aligned} L_{C E}= & {} -\sum _{i=1}^k g_i \log \left( p_i\right) , \end{aligned}$$

where $\varepsilon$ is a small constant to prevent the divisor from being 0. Synthesizing the above formulas, the loss function of RFDDN is

15 $$\begin{aligned} L_{\text{ total } }=\kappa L_{D S C}+\gamma L_{C E}+\lambda L_{\max }. \end{aligned}$$

$\kappa$, $\gamma$, and $\lambda$ are the hyperparameters for balancing each loss function. According to the importance of different losses, we set them to 1, 1, and 0.5, respectively.

## Results and discussion

We introduce the data source and the experimental environment configuration. Then, we compare RFDDN with the state-of-the-art segmentation algorithms and perform ablation experiments. Finally, we analyze and discuss the experimental results.

### Data source and experimental environment

We use two groups of 3D retinal OCT data with the gold standard for experiments. The gold standard is marked by medical professional physicians. The resolution of each 3D data is $1024 \times 512 \times 128$. Each 3D data consists of 128 2D slices, and the resolution of each 2D slice is $1024 \times 512$. The first group includes 43 3D retinal OCT data with the gold standard. The entire dataset contains 5504 2D slices, of which 2816 are training data, 1408 are validation data, and the rest are test data. The second group includes 40 3D retinal OCT data with the gold standard. The entire dataset contains 5120 2D slices, of which 2560 are training data, 1280 are validation data, and the rest are test data. There are four categories of regions in Oct fundus images, namely SRF, PED, retinal edema area (REA), and background. The area of SRF accounts for about 0.7% of the total area, the area of PED accounts for about 0.03% of the total area, the area of REA accounts for about 61% of the total area, and the rest area is the background. The abbreviations that appear in this section are listed in Table 1.

**Table 1:** Abbreviation notes.

Abbreviation	Notes
OCT	Optical coherence tomography
SRF	Subretinal fluid
PED	Pigment epithelial detachment
REA	Retinal edema area
DSC	Dice similarity coefficient
HD95	95th-percentile Hausdorff Distance
ASD	Average symmetric Surface Distance
DS	The model with only deep supervised mechanism
DAB	The model with only double attention block
ARB	The model with only attention refinement block

The hardware environment of experiments was a server with Intel Xeon CPU processor, 16GB memory, and NVIDIA Tesla V100 PCIe GPU (11GB video memory). The visualization, programming, simulation, testing, and numerical calculation processing of experiments were implemented in Python 3.8.

### Evaluation of segmentation performance

We compare RFDDN with the five state-of-the-art OCT image segmentation algorithms, namely U-Net^16^, ReLayNet^20^, CE-Net^25^, MultiResUNet^26^, ISCLNet^8^. We use DSC, 95th-percentile Hausdorff Distance (HD95), Average symmetric Surface Distance (ASD), sensitivity, and specificity as evaluation indicators. The calculation formula of DSC is as follows:

16 $$\begin{aligned} D S C=\frac{2 \times \vert P \cap T \vert }{\vert P \cup T \vert }, \end{aligned}$$

where *P* represents the predicted segmentation result, *T* represents the real segmentation result, $\vert \bullet \vert$ is the cardinality of the set, and $0 \le DSC \le 1$. The larger DSC, the better the segmentation effect. The calculation formula of HD95 is as follows:

17 $$\begin{aligned} H D 95= & {} 95 \% \times \max \left\{ d_{X Y}, d_{Y X}\right\} , \end{aligned}$$

18 $$\begin{aligned} d_{X Y}= & {} \max _{x \in X}\left\{ \min _{y \in Y}\Vert x-y\Vert \right\} , \end{aligned}$$

19 $$\begin{aligned} d_{Y X}= & {} \max _{y \in Y}\left\{ \min _{x \in X}\Vert y-x\Vert \right\} , \end{aligned}$$

where *X* and *Y* respectively represent the point set of the real segmentation result and the point set of the predicted segmentation result, and $\Vert \bullet \Vert$ is a distance function between two points. The smaller HD95, the better the segmentation effect. The calculation formula of ASD is as follows:

20 $$\begin{aligned} A S D=\frac{\sum _{x \in X} \min _{y \in Y}\Vert x-y\Vert +\sum _{y \in Y} \min _{x \in X}\Vert y-x\Vert }{\vert X \vert + \vert Y \vert }. \end{aligned}$$

The smaller ASD, the better the segmentation effect. We use a mini-batch stochastic gradient descent optimization algorithm with momentum to update the network parameters, and set the momentum to 0.9. The gradient threshold is 0.005. The L2 regularization parameter is 1e-4. The epoch is 20, and the number of iterations is 3k. The batch size is 16. The initial learning rate is 1e-3. We reduce the learning rate to 0.1 times of the current learning rate after 1k and 2.5k iterations, respectively. The segmentation results of the six methods on the first group of data are shown in Table 2.

**Table 2:** Segmentation results of the six methods on the first group of data.

Method	DSC	HD95	ASD	Sensitivity	Specificity
U-Net	0.89	0.88	0.36	0.91	0.77
ReLayNet	0.87	0.89	0.38	0.89	0.74
CE-Net	0.92	0.73	0.22	0.94	0.83
MultiResUNet	0.91	0.74	0.25	0.93	0.82
ISCLNet	0.94	0.68	0.17	0.96	0.87
**RFDDN**	**0**.**97**	**0**.**63**	**0**.**13**	**0**.**99**	**0**.**92**

The values obtained by our method are in bold.

RFDDN is superior to the other five methods on all evaluation indicators. DSC, HD95, ASD, sensitivity, and specificity of RFDDN are 0.97, 0.63, 0.13, 0.99, and 0.92, respectively. The results obtained by ISCLNet are the second only inferior to those obtained by RFDDN. DSC, sensitivity, and specificity of RFDDN are 3.2%, 3.1%, and 5.7% higher than those of ISCLNet, respectively. HD95 and ASD of RFDDN are 7.4% and 23.5% lower than those of ISCLNet, respectively. The segmentation results of RFDDN before and after feature discretization on the first group of data are shown in Table 3.

**Table 3:** Segmentation results of RFDDN before and after feature discretization on the first group of data.

Method	DSC	HD95	ASD	Sensitivity	Specificity
UnFD-Net$^{\text{a}}$	0.95	0.65	0.15	0.97	0.89
**RFDDN**	**0**.**97**	**0**.**63**	**0**.**13**	**0**.**99**	**0**.**92**

$^{\text{a}}$UnFD-Net is a model that has the same network structure as RFDDN but lacks the feature discretization module. The values obtained by our method are in bold.

DSC, sensitivity, and specificity of RFDDN are 2.1%, 2.1%, and 3.4% higher than those of UnFD-Net, respectively. HD95 and ASD of RFDDN are 3.1% and 13.3% lower than those of UnFD-Net, respectively. The segmentation results of the six methods on the second group of data are shown in Table 4.

**Table 4:** Segmentation results of the six methods on the second group of data.

Method	DSC	HD95	ASD	Sensitivity	Specificity
U-Net	0.86	0.89	0.37	0.88	0.72
ReLayNet	0.85	0.89	0.38	0.88	0.7
CE-Net	0.88	0.76	0.28	0.91	0.75
MultiResUNet	0.87	0.77	0.29	0.9	0.73
ISCLNet	0.92	0.69	0.19	0.95	0.82
**RFDDN**	**0**.**95**	**0**.**65**	**0**.**16**	**0**.**97**	**0**.**89**

The values obtained by our method are in bold.

RFDDN is superior to the other five methods on all evaluation indicators. DSC, HD95, ASD, sensitivity, and specificity of RFDDN are 0.95, 0.65, 0.16, 0.97, and 0.89, respectively. The results obtained by ISCLNet are the second only inferior to those obtained by RFDDN. DSC, sensitivity, and specificity of RFDDN are 3.3%, 2.1%, and 8.5% higher than those of ISCLNet, respectively. HD95 and ASD of RFDDN are 5.8% and 15.8% lower than those of ISCLNet, respectively. The segmentation results of RFDDN before and after feature discretization on the second group of data are shown in Table 5.

**Table 5:** Segmentation results of RFDDN before and after feature discretization on the second group of data.

Method	DSC	HD95	ASD	Sensitivity	Specificity
UnFD-Net	0.93	0.67	0.18	0.96	0.84
**RFDDN**	**0**.**95**	**0**.**65**	**0**.**16**	**0**.**97**	**0**.**89**

The values obtained by our method are in bold.

**Figure 6:**
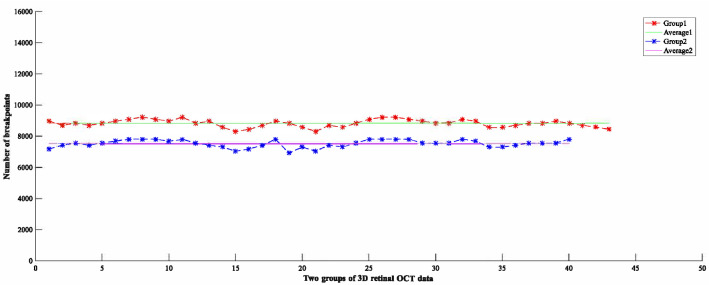
Rough fuzzy set based discretization results on the two groups of 3D retinal OCT data.

DSC, sensitivity, and specificity of RFDDN are 2.2%, 1%, and 6% higher than those of UnFD-Net, respectively. HD95 and ASD of RFDDN are 3% and 11.1% lower than those of UnFD-Net, respectively. The results show that the feature discretization based on the rough fuzzy set can improve the segmentation precision of the deep neural network. The rough fuzzy set based discretization results of two groups of 3D retinal OCT data are shown in Fig. 6.

**Figure 7:**
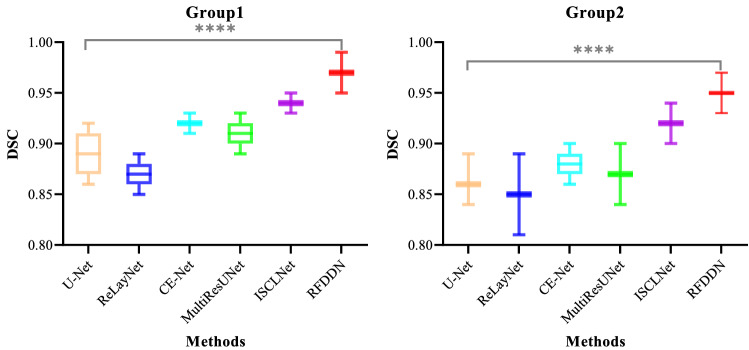
Significance of DSC on the two groups of 3D retinal OCT data.

The number of breakpoints and data inconsistency are two major rough fuzzy set based discretization evaluation indicators. The smaller the number of breakpoints and the data inconsistency, the better the discretization effect. Each 3D retinal OCT data consists of 128 2D slices. The brightness value of each 2D slice is 8 bits (ranging from 0 to 255), and the number of breakpoints is 256. Thus, the initial number of breakpoints for each 3D retinal OCT data is 32768. After feature discretization based on the rough fuzzy set, the average number of breakpoints in the first group is 8826, which is reduced by 73.1%, and the average number of breakpoints in the second group is 7520, which is reduced by 77.1%. The overall data scale decrease by 75.1%. The data inconsistency of both groups is 0. Therefore, the computational efficiency of the model is improved. The significance of DSC on the two groups of 3D retinal OCT data is shown in Fig. 7.

We use one-way ANOVA to analyze the significance of DSC between the six methods on the two groups of 3D retinal OCT data. The threshold of significance level is 0.05. In the box plot, the lines represent the median, 25th, and 75th percentiles. **** indicates $P < 0.0001$. The *P* value is less than 0.05, which indicates a statistically significant difference of DSC between the six methods on the two groups of 3D retinal OCT data.

### Ablation experiment

The initial learning rate is an important hyperparameter of RFDDN. The segmentation results of RFDDN under different initial learning rates are shown in Table 6.

**Table 6:** Segmentation results of RFDDN under different initial learning rates.

Initial learning rate	DSC	HD95	ASD
0.1	0.9632	0.6395	0.1382
0.01	0.9637	0.6388	0.1369
**0**.**001**	**0**.**9682**	**0**.**6327**	**0**.**1347**
0.0001	0.9618	0.6406	0.1397

The values obtained by the optimal initial learning rate are in bold.

An initial learning rate that is extremely large will cause the gradient to oscillate around the minimum, while an initial learning rate that is extremely small will result in slow convergence. RFDDN can achieve the best evaluation indicator values when the initial learning rate is 0.001. The evaluation indicator values of RFDDN under different learning rates have little difference. Therefore, RFDDN is not sensitive to the initial learning rate. Then, we conduct the ablation experiment with four different models. The four models are (a) the baseline with only encoder and decoder; (b) the model with only deep supervised mechanism (DS); (c) the model with only double attention block (DAB); (d) the model with only attention refinement block (ARB). These models have the same pre-trained weights as RFDDN. The segmentation results of RFDDN and the four models are shown in Table 7.

**Table 7:** Results of the ablation experiment.

Method	DSC	HD95	ASD
Baseline	0.78	1.31	0.5
DS	0.81	1.13	0.36
DAB	0.82	1.06	0.32
ARB	0.9	0.76	0.28
**RFDDN**	**0**.**97**	**0**.**63**	**0**.**13**

The values obtained by our method are in bold.

**Figure 8:**
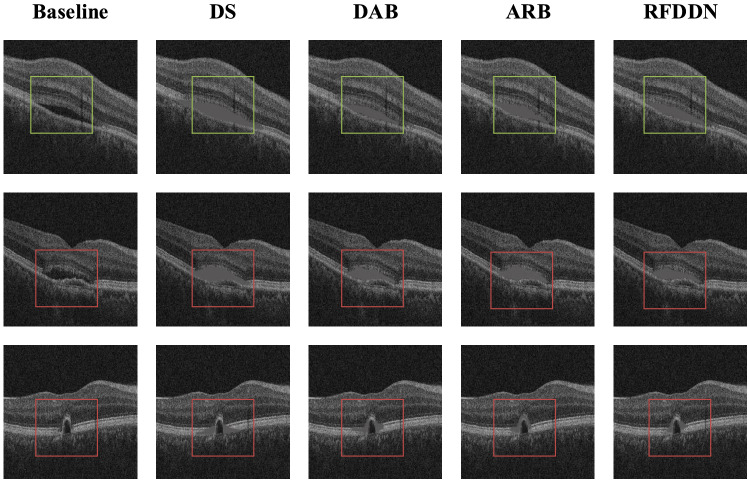
Visual segmentation results of different models.

The baseline has the worst DSC, HD95, and ASD at 0.78, 1.31, and 0.5, respectively. Although DS can alleviate the negative impact of data imbalance, the performance of the model with only DS is still unsatisfactory, and the obtained DSC, HD95, and ASD are 0.81, 1.13, and 0.36, respectively. The introduction of the attention mechanism enables both the model with only DAB and the model with only ARB to obtain better segmentation results than those of the baseline and the model with only DS. DSC of RFDDN is 7.8% better than that of the model with only ARB. HD95 and ASD of RFDDN are 17.1% and 53.6% lower than those of the model with only ARB, respectively. RFDDN has a strong ability to capture contextual information. Therefore, RFDDN can obtain the best segmentation results. The visual segmentation results obtained from the ablation experiment are shown in Fig. 8.

The baseline has the worst segmentation effect. Owing to the lack of ability to capture information, the model with only DS has the poor segmentation ability, which is prone to false segmentation. Although the segmentation performance of the model with only DAB and the model with only ARB has improved, it is still difficult to segment some regions with blurred boundaries. Baseline, DS, DAB, and ARB have artifacts in the area surrounded by green lines, and are not able to clearly display some detail characteristic of slices in the area surrounded by red lines. Compared with these four models, RFDDN generates smaller fuzzy regions. Obviously, RFDDN has the best segmentation effect.

### Discussion

U-Net combines low-level detail information with high-level semantic information by concatenating feature maps from different levels to improve segmentation accuracy. However, U-Net is prone to overfitting during training because of shallower layers and fewer parameters. Furthermore, the consecutive pooling and strided convolutional operations led to the loss of some spatial information. ReLayNet uses a contracting path of encoders to learn a hierarchy of contextual features, followed by an expansive path of decoders for semantic segmentation. However, the convolutional blocks employed by these encoders and decoders have limited ability to capture important features. CE-Net adopts the pre-trained ResNet block in the feature encoder and integrates the dense atrous convolution block and the residual multi-kernel pooling into the ResNet modified U-Net structure to capture more high-level features and preserve more spatial information. Although CE-Net can achieve better segmentation accuracy than that of U-Net, it still faces the problem that the adopted convolutional block has the limited ability to capture important features. MultiResUNet uses Res paths to reconcile the two incompatible sets of features from the encoder and the decoder, and designs MultiRes blocks to augment U-Net with the ability of multi-resolutional analysis. However, it still suffers from the loss of spatial information caused by the consecutive pooling and strided convolutional operations. ISCLNet learns the intra-slice fluid-background similarity and the fluid-retinal layers dissimilarity within an OCT slice, and builds an inter-slice contrastive learning architecture to learn the similarity among adjacent OCT slices. However, it relies on complete OCT volumes that may be difficult to access in the clinic. In addition, none of the above methods has the special mechanism for dealing with noise and uncertain information. RFDDN introduces a deep supervised attention mechanism into the network, and greatly eliminates the negative impact of redundant information and noise through feature discretization based on the rough fuzzy set. Therefore, RFDDN can achieve higher segmentation accuracy.

## Conclusion

Deep learning technology has achieved promising results in optical coherence tomography (OCT) fundus image segmentation. However, the noise and redundant information in the images are the main reasons for the performance bottleneck of the deep network. In addition, the deep network lacks robustness and interpretability, and is difficult to deal with the uncertain information. To this end, we have proposed a deep network embedded with rough fuzzy discretization (RFDDN) for OCT fundus image segmentation. Our contributions are as follows: (1) we establish the information decision table of OCT fundus image segmentation, and calculate the membership degrees of pixels to each segmentation region using the fuzzy c-means clustering to achieve the fuzzification of pixel categories; (2) we design the individual fitness function based on the rough fuzzy set, and use a genetic algorithm to search for the best breakpoints to discretize the features of OCT fundus images to reduce the uncertainty caused by noise and redundant information; (3) we take the feature discretization based on the rough fuzzy set as the pre-module of the deep neural network, and introduce the deep supervised attention mechanism to obtain the important multi-scale information, thereby improving the segmentation accuracy of OCT fundus images. We compare RFDDN with U-Net, ReLayNet, CE-Net, MultiResUNet, and ISCLNet on the two groups of 3D retinal OCT data. RFDDN is superior to the other five methods on all evaluation indicators. The results obtained by ISCLNet are the second only inferior to those obtained by RFDDN. DSC, sensitivity, and specificity of RFDDN are evenly 3.3%, 2.6%, and 7.1% higher than those of ISCLNet, respectively. HD95 and ASD of RFDDN are evenly 6.6% and 19.7% lower than those of ISCLNet, respectively. Then, we compare the results before and after feature discretization. DSC, sensitivity, and specificity of RFDDN are evenly 2.2%, 1.6%, and 4.7% higher than those of UnFD-Net, respectively. HD95 and ASD of RFDDN are evenly 3.1% and 12.2% lower than those of UnFD-Net, respectively. Furthermore, we analyze the hyperparameters of the network and conduct the ablation experiment with four different models. The experimental results show that RFDDN can effectively eliminate the noise and redundant information in Oct fundus images, and greatly improve the accuracy of OCT fundus image segmentation while taking into account the interpretability and computational efficiency.

The future research work includes: (1) applying this method to other medical image datasets to test and improve the adaptability of the model; (2) comparing the proposed method with the state-of-the-art feature discretization algorithms on performance to optimize the feature discretization module, thus improving the generalization ability of the network.

## Data Availability

The datasets used and/or analysed during the current study available from the corresponding author on reasonable request.
